# Endogenous opioid system dysregulation in depression: implications for new therapeutic approaches

**DOI:** 10.1038/s41380-018-0117-2

**Published:** 2018-06-28

**Authors:** Marta Peciña, Jordan F. Karp, Sanjay Mathew, Mark S. Todtenkopf, Elliot W. Ehrich, Jon-Kar Zubieta

**Affiliations:** 1 Department of Psychiatry, University of Pittsburgh, Pittsburgh, PA USA; 2 Menninger Department of Psychiatry & Behavioral Sciences, Baylor College of Medicine, Houston, TX USA; 3 Alkermes, Inc, Waltham, MA USA; 4 Department of Psychiatry, University of Utah Health Sciences Center, Salt Lake City, UT USA

**Keywords:** Depression, Neuroscience

## Abstract

The United States is in the midst of an opioid addiction and overdose crisis precipitated and exacerbated by use of prescription opioid medicines. The majority of opioid prescriptions are dispensed to patients with comorbid mood disorders including major depressive disorder (MDD). A growing body of research indicates that the endogenous opioid system is directly involved in the regulation of mood and is dysregulated in MDD. This involvement of the endogenous opioid system may underlie the disproportionate use of opioids among patients with mood disorders. Emerging approaches to address endogenous opioid dysregulation in MDD may yield novel therapeutics that have a low or absent risk of abuse and addiction relative to µ-opioid agonists. Moreover, agents targeting the endogenous opioid system would be expected to yield clinical benefits qualitatively different from conventional monaminergic antidepressants. The development of safe and effective agents to treat MDD-associated endogenous opioid dysregulation may represent a distinct and currently underappreciated means of addressing treatment resistant depression with the potential to attenuate the on-going opioid crisis.

## Introduction

In 2017, the World Health Organization classified depression as the single largest contributor to global disability worldwide (7.5% of all years lived with disability), with over 300 million affected. It is estimated that prevalence has increased over 18% between 2005 and 2015 [1]. This increase represents the chronicity of the disorder: when people become depressed, cure is elusive, and the condition often follows a relapsing and recurring natural history.

Major depressive disorder (MDD) is composed of low mood, diminished capacity to experience enjoyment, weight and sleep alterations, fatigue, negative assessments of self, cognitive dysfunction with notable difficulties with concentration and decision-making, and recurrent thoughts of death or suicide [2]. Depression becomes more treatment resistant with subsequent episodes, with 50% of those recovering from a first episode having an additional episode, and 80% of those with two or more episodes having another recurrence [3]. Response rates (more than 50% symptomatic improvement) even in community samples and treated open-label with antidepressants, is only reached in 50% of participants, while full remission (more than 75% symptomatic improvement) is only achieved in 30–35% of individuals using first-line antidepressants (serotonin-selective reuptake inhibitors—SSRI’s) [4]. For patients who are non-responsive to two interventions (SSRI and cognitive behavioral therapy or adjuvant treatment), remission rates with subsequent therapy only range from 10 to 25% [5].

Over the last 60 years, there has been minimal progress in bringing antidepressants with novel mechanisms of action from the laboratory to the clinic. Since the introduction of tricyclic antidepressants in the 1950s, virtually all FDA-approved antidepressants inhibit the metabolization of serotonin, norepinephrine, or both. Some exceptions achieve similar biochemical results through inhibitory presynaptic receptor blockade, or varying degrees of postsynaptic receptor activation. Given the inadequate results observed in both controlled trials and in clinical practice with currently available pharmacotherapeutics, there is an urgent need to explore novel therapeutic targets.

Complicating the treatment of MDD and contributing to its chronicity are its frequent comorbidity with anxiety disorders [6] and elevated medical comorbidity [7]. Deliberate use of opioid agonists to self-medicate symptoms of depression is likely a substantial contributor to the current opioid crisis. More than half of all opioid prescriptions for pain in the United States are written for people with comorbid depression and anxiety—i.e., the 16% of Americans who have mood disorders receive 51% all opioids prescribed in the United States [8].

Here we develop the premise that targeting the endogenous opioid system may offer an opportunity to improve outcomes for therapeutically complex patients not responding adequately to currently available antidepressants. While the use of opioid agonists for the treatment of melancholic depression dates back millennia [9, 10]; overdose and safety risks have profoundly limited opioid drug development for depression. This review summarizes current animal and human literature supporting the implication of the opioid system in the regulation of functions thought to be disrupted in, and at the core of, depressive symptomatology, such as alterations in stress responses, anxiety, social bonding, and hedonic and appetitive behaviors. This evidence has energized interest in modulating the endogenous opioid system in an effort to treat MDD and its comorbid conditions, including suicidal ideation. Furthermore, recent translational and clinical efforts posing novel mechanisms to reduce risk of abuse while maintaining clinical efficacy, are starting to show promising results and have the potential to advance the treatment of opioid dysregulation across psychiatric conditions.

## Endogenous opioid pharmacology

**Fig. 1:**
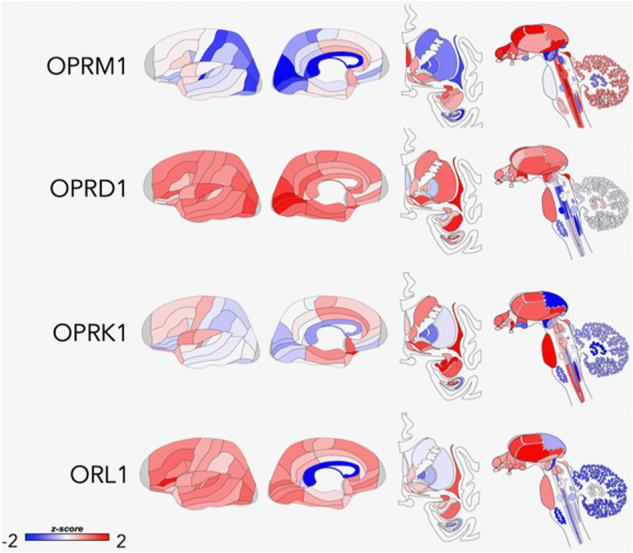
Areas of opioid receptor gene expression (μ = OPRM1; δ = OPRD1; κ = OPRK1; NOP = ORL1) in the human brain (Donor: H0351.1015, 55 yrs, Male, White or Caucasian). The cortical gene expression patterns are displayed on an inflated cortical surface (outer and inner surfaces of the left hemisphere). Subcortical structures of the brain are represented from the frontal view, and subcortical as well as brainstem structures are shown in the side view. The color bar displays expression values using *z*-score normalization. Allen Institute; http://www.brain-map.org

The endogenous opioid system comprises a family of peptides known as β-endorphin, the enkephalins, dynorphins, and their G-protein-coupled receptors known as µ, δ, and κ, and the non-opioid receptor, nociceptin (NOP), previously referred to as opioid receptor-like 1 receptors. β-endorphin, as well as drugs similar to morphine, act primarily at µ-opioid receptors. The naturally occuring Met- and Leu-enkephalins have high affinity for δ-opioid receptors, but also high affinity for µ-opioid receptors. The endogenous peptide dynorphin, as well as peptides related to dynorphin, primarily act on κ-opioid receptors. Nociceptin/orphanin FQ is the endogenous peptide for NOP receptors [11]. Furthermore, both human [12–17] and rodent studies [18–21] have characterized the expression of these peptides and their receptors in limbic and paralimbic regions centrally involved in the modulation of affective states, neuroendocrine and autonomic stress responses, and mood and motivational processes. These processes are disregulated in MDD in the anterior cingulate cortex (ACC), prefrontal cortex, medial thalamus, anterior hypothalamus, nucleus accumbens, amygdala, periaqueductal gray, and ventral tegmental area (Fig. 1), and as such are a logical target for drug development (see ref. [9] for a review). Additionally, opioid peptides are expressed both in the central and peripheral nervous systems [22], and the endogenous opioid system has critical roles in several physiological functions such as pain processing, response to and regulation of stress, gastrointestinal transit, respiration, endocrine, and immune functions [23].

Activation of µ-opioid receptors is primarily known for their analgesic effect. In addition, several lines of evidence have demonstrated a role of µ-opioid receptor function in the regulation of behaviors important for the success of species such as appetite and reproduction [24–26]. It is also centrally involved in responses to social stimuli, whether modulating the distress of social rejection [27, 28] or mediating positive responses to social acceptance and affiliative behavior [29–31]. δ-opioid receptors also have a role in pain modulation [32–35]. In addition, κ-opioid receptors are associated with pain modulation [36–38], and of particular interest in peripherally mediated nociception such as pruritus [39]. In nonclinical studies, activity at NOP receptors has been associated with pain mechanisms and several behaviors linked to psychological stress [40, 41].

## Preclinical evidence of opioid system involvement in depression

Most of the studies discussed below utilize a paradigm of behavioral despair known as the forced swim test. In this paradigm, rats (or mice) are placed in a narrow, inescapable cylinder of water. At first there is vigorous activity that ceases and the rat only does the necessary movements to keep the head above water. This immobility is interpreted to be a state of behavioral despair, and that the rat has learned escape is impossible. The immobility time is indicative of a depressive-like effect in that most drugs that have antidepressant effects in humans reduce immobility time [42].

Preclinical evidence has suggested that activation of µ-opioid receptors has antidepressant-like effects [43, 44]. In mice, opioids (morphine, codeine, levorphanol, methadone, and tramadol) decreased immobility in a tail suspension test [43] (another commonly used rodent assay similar in concept to the forced swim test). In another study, utilizing mice in the forced swim test, both morphine and agmatine (an endogenous aminoguanidine) decreased immobility time and these effects were blocked by pretreatment with naloxone (a µ-opioid receptor antagonist) [45]. In rats, buprenorphine (a partial µ-opioid receptor agonist) also reduces immobility [46, 47]. In addition, the role of β-endorphins in the pathophysiology of MDD has been reviewed [48]. Interestingly, it has been reported that naltrexone (an opioid receptor antagonist) enhances the effects of antidepressants in both the forced swim test and the tail suspension test as well as a foot shock-induced behavioral despair paradigm [49]. The reason or mechanism by which this occurs is currently unknown and suggest a complex system that requires further study.

Although primarily limited to preclinical data that has yet to translate to the clinic, activity at δ-opioid receptors may also have antidepressant-like effects. In one of the earlier studies examining the role of this system, administration of exogenous enkephalins had antidepressant-like effects in the forced swim test [50]. Furthermore, in one of the first studies examining the role of δ-opioid receptors, δ-opioid receptor-null mice exhibited depressive-like behaviors [51]. Both the administration of enkephalinase inhibitors, which would increase the synaptic concentrations of enkephalins, as well as direct δ-opioid receptor agonists induce antidepressant-like effects in animal models [52]. All this has led to the hypothesis that deficits in these mechanisms may be implicated in the pathophysiology of depression, potentially through their effects on the mesolimbic dopamine system that is associated with the rewarding effects of food and sex, and more recently has also been associated with depression [53]; however, direct evidence is lacking in humans. Lastly, increases in brain derived neurotrophic factor (BDNF) mRNA expression in rat frontal cortex, hippocampus, and basolateral amygdala have been observed after a single administration of a δ-opioid receptor agonist [54], a mechanism that appears critical in the response to antidepressants through their effects on neuronal BDNF levels and BDNF-mediated neuroplasticity [55, 56].

It has been established that activation of κ-opioid receptors produces aversive and depressive-like states in humans [57] opposite to that of µ- and δ-opioid receptor activation. In addition, the depressive-like effects of a κ-opioid receptor agonist have also been characterized both behaviorally and neurochemically in rats [58]. In preclinical studies, κ-opioid receptor activation increases immobility in the forced swim test [59] and elevates brain reward thresholds [60, 61], indicative of an anhedonic depressive-like effect. Conversely, administration of a putative κ-opioid receptor antagonist reverses these effects indicative of an antidepressant-like effect [59, 61]. Additional preclinical studies have also demonstrated the ability of κ-opioid receptor antagonists to have antidepressant-like effects [62] as well as reduce repeated forced swim stress-induced immobility [63] and decrease anhedonia-like responses in a cocaine withdrawal paradigm [64]. Together, these data suggest a potential utility of κ-opioid antagonists in the study and treatment of depression [58].

As noted above, recent studies have begun to elucidate the role of NOP receptors in mediating mood, and are exploring the utility of NOP antagonists for depression [65]. NOP and N/OFQ are located in areas that are crucial to mood control including but not limited to amygdala, hippocampus, thalamus, and cortical processing areas [66]. There is now good evidence from animal work for a role for the N/OFQ–NOP system in emotional disorders [66] including anxiety [67] and depression [68, 69]. For example, NOP receptor antagonists reduce immobility time in mice in the forced swim test [68], and NOP receptor knockout mice display an antidepressant-like phenotype in the forced swim test [70]. In preclinical studies examining the novel NOP antagonist LY2940094, there was a transient increase in prefrontal serotonin concentrations as well as a dose-dependent reduction in immobility in the forced swim test [71]. Together, both results are similar to the effects of known SSRIs approved for the treatment of depression.

## Human evidence of opioid regulation of mood

There are well-known species differences in the distribution of opioid receptors in the brain. In general, there is relatively less δ-opioid receptor binding in the human brain compared to the rat brain, and relatively more κ-opioid receptor binding [72]. As such, it is prudent to be careful in extrapolating results from rodent data to humans, and human mechanistic studies are highly desirable.

**Fig. 2:**
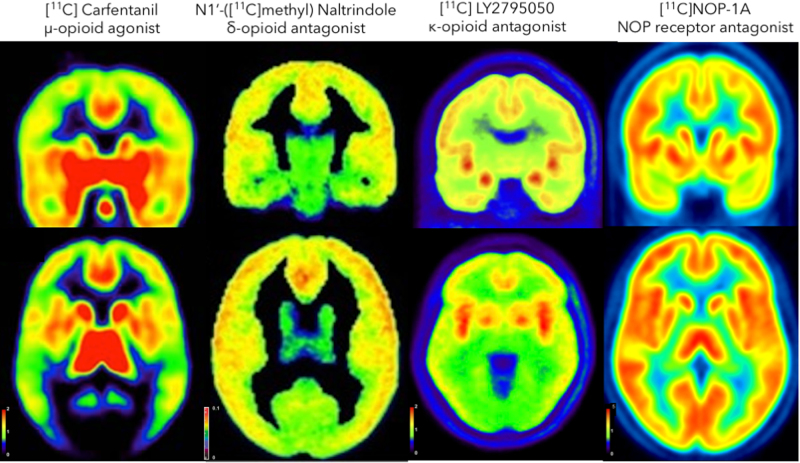
Positron emission tomography (PET) baseline measures of opioid receptor binding in humans [images averaged across a group of subjects (*n* < 20 for all groups)]. Images are color-coded according to the scale shown so that highest concentrations of the radiotracer are represented by red and lowest concentrations by black/purple. Binding maps in the coronal (top) and axial (bottom) view show greatest binding in the striatum and insular cortex for all radiotracers, except for the δ-opioid receptor antagonist: N1′-([11 C]methyl) Naltrindol. Left: μ-opioid receptor agonist: [^11^C]; Carfentanil; δ-opioid receptor antagonist: N1′-([^11^C]methyl) Naltrindol; κ-opioid receptor antagonist: [^11^C] LY2795050; nociceptin receptor: [^11^C]NOP-1A. Reproduced with permission [137–139]. NOP receptor agonist: [^11^C]NOP-1A, images provided by Rajesh Narendran

A number of different approaches have been used to investigate the mechanisms underlying opioid receptors and function in humans. Among them, the use of selective radioligands and positron emission tomography (PET) (Fig. 2), as well as genetic and pharmacological approaches, have resulted in major contributions to the field, particularly as it relates to the processing of emotions and social cues. These measures show receptor availability under baseline conditions, which reflects their concentration, minus receptor occupancy by the endogenous ligand—which for endogenous opioid systems is thought to be very low. In addition, PET studies involving experimental challenges have allowed for the quantification of neurotransmitter release. Under these kinds of experimental conditions, reductions in in vivo receptor availability after an acute challenge are thought to reflect neurotransmitter release and competition between the radiotracer and the endogenous ligand for the receptor sites, providing an indirect measure of presynaptic function.

µ-Opioid receptors are widely distributed in the brain, and their location ostensibly overlaps with regions implicated in emotion regulation [14]. The µ-opioid receptor-selective radiotracer [^11^C]carfentanil has been commonly used to investigate the link between opioid neurotransmission and emotion regulation. In initial studies, Zubieta et al. used in vivo measures of µ-opioid receptors during a sadness induction paradigm, a stimulus, which does not activate objective measurements of stress (i.e., cortisol or ACTH release) but induces a temporary low mood state. This emotional challenge was associated with reductions in endogenous opioid neurotransmission in a widespread network of regions implicated in emotion regulation [73], which were associated with increases and reductions in negative and positive affect, respectively.

Several studies have linked baseline measures of µ-opioid receptor availability to the prediction of antidepressant treatment response. For example, Zubieta and colleague [74] found that reductions in µ-opioid receptor availability were associated with poor treatment response to an SSRI, as well as higher plasma levels of stress hormones (cortisol and ACTH), while an exaggerated sadness-induced opioid release in the rostral anterior cingulate cortex (ACC)-predicted SSRI non-response. Similar sadness-induced exaggerated responses in the rostral ACC were also observed in patients with borderline personality disorder [75], a clinical diagnosis characterized by severely disrupted affective processing and typically poor response to existing antidepressant medications.

In a later study, the same group investigated the role of opioid neurotransmission in the formation of placebo responses in patients with MDD [76]. This investigation followed-up on growing evidence linking the opioid system to placebo analgesia [77–80]. This study involved two placebo lead-in phases followed by an open antidepressant administration. The two oral placebos were identical, but described as having either active or inactive fast-acting antidepressant-like effects. Patients were studied with PET and the μ-opioid receptor-selective radiotracer [^11^C]carfentanil after each 1-week inactive and active oral placebo treatment. In this sample, reduced baseline µ-opioid receptor availability in the nucleus accumbens predicted a lack of response to SSRI antidepressant medication [76]. Furthermore, the capacity to activate endogenous opioid neurotransmission in response to expectations of improvement elicited by the administration of the oral placebo, predicted the response to both oral placebo and antidepressant treatments, explaining up to 40% of the variance in treatment responses. This evidence suggests that µ-opioid receptors are not only involved in the neurobiology of normal and pathological emotional, hedonic, and stress processing, but also the response to both pharmacological and cognitive mechanisms of treatment response.

**Fig. 3:**
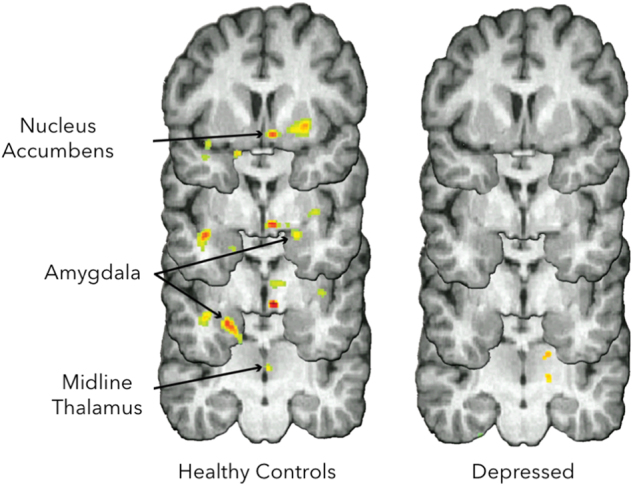
Measure of changes in μ-opioid receptor availability in vivo with positron emission tomography (PET) during social rejection (not being liked by others) and acceptance (being liked by others). Compared to depressed patients, healthy controls showed greater rejection-induced opioid release in the nucleus accumbens, amygdala and midline thalamus. Reproduced with permission [82]

In addition, human neuroimaging studies have established a link between opioid neurotransmission and the processing of social cues. Initial evidence suggested that social rejection and physical pain shared similar neural pathways [81]. These studies supported the hypothesis that the µ-opioid receptor system could be involved in regulating other forms of non-painful stressor (i.e., social “pain”). This hypothesis was first tested in healthy volunteers using a social feedback task in response to social rejection and acceptance cues and the quantification of regional µ-opioid receptor availability. Greater opioid release in regions involved in emotion regulation during social rejection was significantly associated with higher scores in resiliency traits as well as reduced negative affect, consistent with an adaptive role of endogenous opioid neurotransmission on these processes [28, 82]. Not surprisingly, in a follow-up study, patients with depression, compared to controls, had reduced opioid release in similar regions [82]. This evidence suggests that the endogenous opioid system, in particular μ-opioid receptors, has a key role in the processing of social cues which seems to be particularly altered in patients with MDD (Fig. 3).

At the genetic level, several studies have investigated the relationship between variations within the human μ-opioid receptor gene (*OPRM1*) and depression-related traits and symptoms. The best studied genetic variant in the *OPRM1* gene is a single-nucleotide polymorphism that changes the amino acid at position 40 in the N-terminal domain of the receptor from asparagine to aspartate [Asn40Asp, A118G, rs1799971 [83]]. Animal studies have suggested that the G118 allele is associated with loss of function of the receptor, lower surface receptor expression, decreased forskolin-induced cAMP activation, and lower agonist-induced MOPR activation [84, 85]. In a human PET study, 118G allele carriers (G-carriers), compared to A/A homozygotes, had an overall brain reduction of baseline μ-opioid receptor availability in regions implicated in pain and affective regulation. G-carriers also reported higher trait neuroticism and depression scores, which were inversely correlated with the in vivo brain measures of receptor concentrations [86]. G-carriers have also shown blunted cortisol responses to stressors, but greater cortisol responses to naloxone administration, suggesting differences in receptor affinity in G allele carriers [87]. Furthermore, G-carriers had greater reactivity to social rejection in the dorsal ACC and anterior insula, where the dorsal ACC activity in response to social rejection further mediated the relationship between the A118G polymorphism and dispositional sensitivity to rejection [88]. Therefore, G-carriers, possibly through a lower expression of µ-opioid receptors and a reduced capacity to release endogenous opioids, may have greater vulnerability for depressive-like symptoms and poorer treatment responses to SSRI treatment [74, 86].

Despite strong preclinical evidence, little is known about the role of δ-, κ-, and NOP receptors in the neurobiology and the mechanisms involved in the response to treatment in mood disorders. The localization of δ-opioid receptors in the amygdala is consistent with their modulation of fear and anxiety states [89], whereas localization in the cortex and hippocampus is consistent with their potential antidepressant effects [54]. On the other hand, and consistent with its role regulating reward, pain, and emotional processing, κ-opioid receptors are present in the deep layers of cortical regions and in the striatum, hippocampus, amygdala, and thalamus [90], where NOP receptors are also localized [66]. However, the lack of availability of specific δ-, κ- and NOP- agonists or antagonists for human administration, as well as the limited availability of selective radiotracers, has limited the understanding of these systems in clinical populations. A selective δ-opioid receptor antagonist [[^11^C]-methyl-naltrindole [91]] and several selective κ-opioid receptor radioligands (e.g., agonist ligands: [^11^C]-GR89696, [^11^C]-GR103545; antagonist ligands: [^11^C]-MeJDTic, [^11^C]-LY2795050 or [^11^C]-LY2459989) [92], are available for human use, but yet have not been applied to mood disorders. The failure of initial proof-of-concept clinical studies using δ-opioid receptor agonists [51], as well as a higher risk of producing convulsions [51], might have discouraged clinical mechanistic studies. Similarly, the use of the NOP receptor antagonist radiotracer [^11^C] (S)-3-(2′-fluoro-6′,7′-dihydrospiro[piperidine-4,4′- thieno[3,2-c]pyran]-1-yl)-2-(2-fluorobenzyl)-*N*-methylpropanamide (NOP-1A) has been successfully validated for use in human PET studies [93, 94], as well as clinical populations [95]. Still, the mechanisms through which NOP receptors modulate mood or anxiety disorders in humans, as suggested in clinical studies [71, 96] are currently unknown.

## Clinical evidence in MDD

The initial clinical studies to formally evaluate opioidergic agents in the treatment of depression took place during the 1970s and early 1980s, just a few years following the initial identification of the endogenous opioid peptides [97, 98]. At least four studies were conducted utilizing intravenous infusions of various doses of synthetic endorphin peptide preparations [99–102]. Two additional studies evaluated synthetic opioids [103, 104]. Overall, the majority of subjects in these clinical trials experienced substantial improvements in depressive symptoms within hours of administration. The most convincing evidence of a significant improvement in depressive systems was reported from a placebo-controlled crossover study of a single intravenous dose of β-endorphin in ten subjects with either unipolar or bipolar depression [100]. Interpretation of these early studies is limited by small patient sample, limited controls, brief duration of dosing, and no probes of mechanism, including CNS penetration, to confirm clinical observations. However, as a composite, they represent the first formal experimental assessment of the “opium cure” following centuries of its use based on empiric experience.

In subsequent decades, there have been numerous clinical studies evaluating buprenorphine in the treatment of depression [105–110]. Buprenorphine is a µ-opioid receptor partial agonist and thus offers potential safety advantages compared with a full µ-opioid agonist [111]. In addition to its activity at µ-opioid receptors, buprenorphine is a κ-opioid receptor antagonist and may confer antidepressant activity by blocking this receptor [58]. Although only one included a placebo control [112], these studies uniformly reported substantial clinical improvements in patients with treatment-resistant depression, including patients who were unresponsive to electroconvulsive therapy. The mean buprenorphine dose evaluated in these studies was low, sub-euphoric, ranging from 0.2 to 1.2 mg/day. Consistent with this report, low-dose buprenorphine (0.2 mg sublingual), reduced emotional reactivity and improved negative affect in volunteers with a range of depression severity symptoms in a laboratory setting [113]. Direct assessment of drug effects in this study revealed no evidence of drug high or euphoria.

Overall, the clinical experience provides evidence that low-dose buprenorphine may have therapeutic activity in the treatment of depression and that this activity does not require or derive from a frank euphoric effect of the drug.

In addition to antidepressant effects, exposure to opioids may also provide benefit to acutely suicidal patients. In a recent multicenter placebo-controlled study evaluating very low doses of buprenorphine (median dose 0.44 mg/day) in acutely suicidal patients, compared to placebo, buprenorphine led to a significant reduction in suicidality [114]. Effects were observed both in patients with depression or borderline personality disorder and were apparent when buprenorphine was used as either monotherapy or augmentation to standard antidepressant pharmacotherapy.

Despite evidence of the antidepressant activity of opioids and the urgent need for antidepressant agents with novel mechanism of action, the routine use of µ-opioid receptor full and partial agonists in clinical practice is necessarily limited by the potential for abuse and dependence.

To address this tension between dependency and efficacy, and given reports that buprenorphine results in rapid resolution of symptoms [108, 114], dose-finding efforts must be a priority in the study of re-purposed and newly developed opioidergic molecules for neuropsychiatric conditions. In addition to studying clinical effect and safety outcomes, studies must assess changes in physiology (e.g., pupillometry, skin conductance), circuitry (fMRI, magnetoencephalography), molecular activity, and receptor occupancy (PET) at a variety of doses to determine the optimal dose range at which both target engagement and clinical effects are observed.

An alternate strategy to modulate the endogenous opioid system in the treatment of MDD—while avoiding the potential for abuse and dependence—has focused on developing agents that selectively target other, non-µ-opioid receptors that are predicted by animal studies to yield antidepressant activity. Selective δ-opioid receptor agonists [52], nociceptin [71], and κ-opioid receptor antagonists [58] have been introduced into the clinic; however, reports of clinical efficacy with these agents in patients with MDD have yet to appear in the published literature.

An emerging approach designed to address endogenous opioid dysregulation in the context of depression while minimizing opioid abuse and dependence is to simultaneously administer both a µ-opioid receptor agonist and an antagonist with opposing pharmacologic activities of similar magnitude and pharmacokinetics. Co-administration of a µ-opioid antagonist to counteract the µ-opioid agonist effects of an agonist results in a combination with lower intrinsic potential for abuse and dependence. In an animal model, the combination of buprenorphine and naltrexone, in an attempt to reduce the reinforcing effects of µ-agonism and potentiating k-antagonism, resulted in antidepressant-like responses in mice, while eliminating locomotor and rewarding effects [115]. In humans, and using a similar approach, antidepressant activity following daily dosing of buprenorphine combined with samidorphan, a µ-opioid receptor antagonist, has been reported in a small one-week pilot study in patients with MDD with a previous inadequate response to standard antidepressants [116]. In this study, the antidepressant effects observed were greater in patients treated with a 1:1 buprenorphine:samidorphan ratio associated with maximal µ-opioid receptor blockade as compared to a 8:1 ratio associated with partial µ-opioid receptor blockade. This result suggests that greater µ-opioid activity is not necessarily linked to greater antidepressant activity. A follow-up larger phase 2 multi-week clinical study of the 1:1 buprenorphine:samidorphan ratio confirmed the pilot study finding, reporting significant benefits versus placebo [117].

The mechanism of action of the opioid agonist-antagonist combination is not precisely understood and requires further examination. It is possible that very subtle µ-opioid modulation by the combination may be sufficient to ameliorate dysregulated or impaired endogenous opioidergic tone in depressed patients. An alternative, but not mutually exclusive, explanation is that the buprenorphine:samidorphan combination is acting as a functional κ-opioid receptor antagonist as the intrinsic κ-opioid receptor antagonism of buprenorphine is unaffected by samidorphan. Finally, the µ-opioid effects of both the agonist and the opposing antagonist may both contribute and function together to constrain endogenous opioid tone within a desirable range.

## Other CNS disease considerations

### Post-traumatic stress disorder (PTSD)

Beyond the treatment of depression, it is reasonable to consider applications of opioid receptor modulation to the broader range of stress-related psychiatric conditions marked by negative affect, anxiety, social rejection, and altered pain sensitivity. PTSD is a candidate disorder that may benefit from modulation of the opioid system. Indeed, in a recent survey of PTSD researchers, opioid receptor drugs were ranked in the top five therapeutic targets for PTSD worthy of further study [118].

A recent observational study in veterans diagnosed with PTSD, chronic pain, and opioid use disorder found that twice as many veterans who received buprenorphine compared to moderately high-dose opioid therapy experienced improvement in post-traumatic symptoms (PTS) [119]. Tramadol, an atypical analgesic with µ-opioid and non-opioid mechanisms, was found to benefit male veterans with combat-related PTSD [120]. However, given the relatively high rates of chronic pain among veterans [121], a challenge to the interpretation of veteran treatment studies in PTSD is disentangling antinociceptive properties from its other neuropsychiatric effects such as anxiolytic, improved mood, and enhanced resilience to stress. In addition to µ-opioid receptor targets, selective κ-opioid receptor antagonists may provide a neurobiological rational approach for anhedonic symptoms and reward-related dysfunction associated with PTSD and trauma-related conditions. A NIH funded trial of a selective κ-opioid receptor antagonist in patients with a broad range of depressive, anxiety, and trauma-related pathology was recently completed and results are pending (NCT02218736).

Besides investigations in individuals with chronic PTSD, opioids are being used in PTSD prevention trials in at-risk trauma victims. For example, in an animal model of PTSD, morphine prevented the development of stress-enhanced fear learning [122]. Clinically, the use of morphine during early resuscitation and trauma care significantly lowered the risk of PTSD in injured U.S. military personnel [123]. In a similar study with civilians, administering opioids after traumatic injury has been associated with lower rates of PTSD symptoms in prospectively followed samples using a naturalistic design [124, 125].

### Obsessive-compulsive disorder (OCD)

Abnormalities in amygdalo-cortical and cortico-striatal circuitry are established in OCD [126]. These areas of the brain, rich in dopaminergic structures and their anatomical targets as well as opioid receptors, are a rational target for opioid modulation for patients with SSRI-resistant OCD. The prevalence of OCD in opioid-dependent patient samples was found to be four times higher than the general population; and there are reports of OCD symptom worsening during methadone taper [127, 128]. A small placebo and lorazepam-controlled randomized trial in SSRI-resistant OCD found that once-weekly oral morphine administered for 2 weeks was more effective than placebo, while lorazepam was not [129]. There is also open-label evidence for the atypical analgesic tramadol in OCD [130]. While conflicting, these reports suggest abnormal functioning of the opioid system in OCD and repetitive-like behavior syndromes, which by their very nature provide repetitive rewards.

## Other applications

A linkage between endogenous opioid dysfunction and borderline personality disorder has been proposed based on multiple lines of evidence. Evidence includes alterations in plasma levels of opioid peptides, impairment in resiliency and social attachment (i.e., opioid-related behaviors), and a high incidence of opioid dependency among individuals with borderline personality [131, 132]. Moreover, there is a high rate of self-injurious behavior (i.e., “cutting”), a common feature of borderline personality disorder, that is thought to stimulate endogenous opioid release and has been associated with decreased levels of β-endorphin in cerebrospinal fluid [133]. Using PET imaging, Prossin et al. [75] provided confirmation of the borderline personality—endogenous opioid hypothesis by demonstrating significant abnormalities in µ-opioid receptor levels at baseline and exagerated endogenous opioid release following sadness induction in patients with borderline personality disorder, compared to controls. Use of opioid agents to address underlying endogenous abnormalities may represent an important future therapeutic strategy.

Endogenous opioid dysregulation has also been implicated in autism spectrum disorders, which are associated with impairments in social behavior and attachment, repetitive stereotypies, and motor hyperactivity [134]. Therapy with opioid antagonists have improved hyperactivity and restlessness symptoms with unclear effects on other core features of autism such as abnormal social behavior [135]. Further research is needed to identify patient subsets who might best benefit from an endogenous opioid system directed treatment.

## Future directions

Drugs with novel mechanism of action, rapid onset of action, and improved safety profiles are needed for mood, anxiety, and stress-related conditions that have not responded to conventional monoaminergic modulation. It is established that full opioid agonists can induce euphoria and lead to dependence. However, as we noted, the endogenous opioid system is dysregulated and impaired in MDD and has a critical role in motivation, social attachment, and resiliency. Thus, treatment of endogenous opioid dysregulation in MDD has the potential to provide clinical benefits that are distinct and may extend beyond benefits conferred by conventional antidepressants. Clinical studies of very low (i.e., sub-euphoric) doses of opioid agonists, and opioid agonist-antagonist combinations indicate that therapeutic benefit is attainable in the treatment of MDD while minimizing or avoiding abuse liability. Finally, agents that largely bypass µ-opioid receptors and specifically function as either δ-opioid receptor agonists, κ-opioid receptor antagonists, or NOP agonists may produce antidepressant effects without risk of addiction.

Biased opioid receptor ligands represent an emerging area of research. In contrast to existing opioidergic agents, biased opioid ligands bind selectively to activate intracellular G-proteins following receptor engagement, but fail to engage the beta-arresting signaling pathway [136]. Although research in this area remains at an early phase, discovery and development of biased opioid ligands may ultimately yield new therapeutic agents that retain the beneficial therapeutic properties of opioids in the treatment of depression and other psychiatric disorders while minimizing adverse properties such as respiratory depression and abuse potential.

Given the relatively rapid onset of opioids on symptoms of mood and anxiety, other treatment paradigms may also be explored in which these medications are not prescribed long-term, but as “rescue,” “prevention,” and synergistic medications. For example, the short-term use of opioids with κ-opioid receptor antagonism activity in the acute period post-trauma may have a role in preventing chronic PTSD symptoms. In patients who are stress-reactive and hospitalized for suicidal behavior, co-prescribing low-dose buprenorphine along with a monoaminergic agent such as a SSRI may provide immediate relief and reduction in suicidal ideation, allowing time for the clinical effect of the antidepressant to evolve.

Two major public health issues, the opioid addiction epidemic and major depression are linked by underlying endogenous opioid dysregulation. This linkage is manifest in the disproportionate use of opioids by patients with mood disorders who account for the majority of prescription opioid use in the United States. Emerging research is elucidating the mechanisms underlying dysregulation of the endogenous opioid system in depression and other mood disorders. This has led to increased understanding of the shared neural circuitry that mediates the perception of both emotional-social pain and nociceptive pain [27].

Novel pharmacologic approaches based on this research may yield new treatments for depression targeting the endogenous opioid system with low or absent addictive potential. Given the involvement of the endogenous opioid system in social attachment, resiliency, and hedonic tone, these treatments would be expected to confer clinical benefits that are distinct from monoamine-based therapies, particularly in patients who are inadequately responsive to standard antidepressants. Further research is required.

The use of µ-opioid receptor agonists by individuals with mood disorders may reflect either deliberate or inadvertent self-medication of social and emotional pain. This phenomenon would exacerbate the opioid addiction crisis. Ultimately, the development of targeted therapies, with low risk for abuse, to address mood-related endogenous opioid dysregulation would represent a much needed alternative to highly addictive µ-opioid receptor agonists and thereby provide a new and distinct opportunity to contribute to addressing the on-going opioid addiction crisis.
